# Palineo score: development of a score to identify the palliative care needs of neonatal patients admitted to the neonatal intensive care unit

**DOI:** 10.62675/2965-2774.20250039

**Published:** 2025-08-18

**Authors:** Silvia Maria de Macedo Barbosa, Vivian Taciana Simioni Santana, Cibele Regina Laureano Gonçalves, Estéfanie Santana Teixeira Santos, Priscila Endo Takahashi, Pâmella Helena Leme da Silva Costa, Keila da Silva Alves, Cibele Wolf Lebrão, Alex Castro

**Affiliations:** 1 Hospital da Mulher São Bernardo do Campo SP Brazil Hospital da Mulher - São Bernardo do Campo (SP), Brazil.; 2 Autononia: Terapia Ocupacional Ltda São Paulo SP Brazil Autononia: Terapia Ocupacional Ltda - São Paulo (SP), Brazil.; 3 Brazilian Biosciences National Laboratory Brazilian Center for Research on Energy and Materials Campinas SP Brazil Brazilian Biosciences National Laboratory, Brazilian Center for Research on Energy and Materials - Campinas (SP), Brazil.

**Keywords:** Infant, newborn, Palliative care, Neonatal intensive care unit, Quality of life, Decision-making, Surveys and questionnaires

## Abstract

**Objective:**

To develop a score (Palineo score) to identify the palliative care needs of newborn patients admitted to a Brazilian neonatal intensive care unit of a tertiary maternity hospital that serves as a reference center for high-risk pregnancies, ensuring timely follow-up by a specialist.

**Methods:**

Patients were assessed by three specialists using a questionnaire that included the same clinical elements as those used for the Palineo score but did not assign scores to the criteria. The score was determined by the consensus reached by the specialists. A score was subsequently assigned to each component of the Palineo score, allowing for comparisons between the specialists’ assessments and the Palineo score. All the information was retrospectively obtained from the electronic medical records. Data were collected and evaluated on the third and seventh days of life and then weekly until discharge from the neonatal intensive care unit, transfer to another service, or death. The Palineo score was applied to each patient to establish discriminatory cutoff points for the classifications initially assigned by the specialists.

**Results:**

The score showed agreement across the evaluations (k = 0.85). The discriminant values of the Palineo score between patients classified as palliative intent and associated palliative care revealed exceptional accuracy for both the first application of the score (p < 0.001) and the final application (p < 0.001). The discriminant values between patients categorized as associated palliative care and those categorized as specialized palliative care showed exceptional accuracy for the first application (p = 0.005) and final application (p < 0.001) of the score.

**Conclusion:**

Despite exceptional accuracy, discrepant values should be considered with caution. This study should be replicated in a larger sample.

## INTRODUCTION

Advances in obstetrics, fetal medicine, and neonatology have enabled the survival of newborns who were once regarded as nonviable. Maintaining life-sustaining treatments and managing therapeutic limitations still present significant challenges, often causing parents and health care teams to question the course of action. The survival of newborns with life-limiting or life-threatening conditions involves significant suffering related to both mortality and morbidities.^(1–3)^

Prolonging life at all costs is no longer a priority, underscoring the need for patient-centered care plans to prevent devastating consequences for families.^(4,5)^ While withholding or withdrawing life-sustaining treatment alongside palliative care, may be appropriate for some newborns, this is rarely implemented in practice. Selecting patients for neonatal palliative care is challenging, partly owing to the mistaken belief that palliative care hastens death — a misperception shaped by practices in adult palliative care.^(6–8)^

In pediatric palliative care, early referral is crucial for identifying needs and planning interventions. Children may die early or survive for many years, and a holistic approach that takes into account individual values and family wishes is therefore important. Determining the extent of appropriate therapeutic interventions for critically ill newborns is one of the greatest challenges in neonatology.^(5,9)^

To overcome these challenges, we developed an innovative scoring system for neonatal patients. This system identifies candidates for palliative care and classifies them on the basis of clinical and functional criteria, minimizing ambiguity and reducing cultural or personal bias. With this system, health care teams can deliver timely palliative care at intensive care units (ICUs).

## METHODS

This is a retrospective study involving newborns admitted to the neonatal ICU of a tertiary public maternity hospital, a reference center for high-risk pregnancies, in the state of São Paulo, Brazil. The hospital has approximately 420 births per month, approximately 6% of which require intensive care. Among these, 13% are extremely premature, 29% are moderately premature, 17% are late preterm, 2% are full-term, and approximately 20% have some type of malformation. This study was carried out from August 2020 to August 2021 and was approved by the local Research Ethics Committee (CAAE 34722520.7.0000.0082, process no. 4184262). As patient medical records were used for data collection, an informed consent form was not needed.

All newborns admitted to the neonatal intensive care unit during this period were included in the study. The exclusion criteria were newborns who died or had to be transferred within the first 72 hours of their stay in the neonatal intensive care unit, as well as those with a corrected or chronological age over 6 months in cases of prolonged hospital stays.

Initially, the newborns were evaluated and classified as palliative intent, associated palliative intent or specialized palliative intent by pediatric palliative care specialists (two doctors and one physical therapist). A checklist was used for this assessment, with a focus on factors that could affect the neuropsychomotor development and prognosis of the newborn. Clinical and functional criteria routinely collected in clinical practice were used. The classification made by the largest number of experts was considered. If the experts disagreed, a fourth specialist was consulted to make the final decision.

A point distribution system was applied to the clinical criteria for the development of the Palineo score (Table 1), which is based on the degree of impairment, presence of comorbidities, and potential impact on prognosis in the short, medium, and long term. Seven clinical criteria were selected for evaluation, with the higher scores corresponding to greater impact on the newborn's life (mild: 0 - 3, moderate: 4 - 6, severe: 7 - 9, and very severe: 10). The following variables were evaluated: (I) gestational age, (II) birth weight, (III) life-threatening conditions, (IV) severe central nervous system conditions, (V) use of vasoactive drugs, (VI) need for ventilatory support, and (VII) artificial means of ventilation and feeding. A lower gestational age and birth weight are associated with a greater risk of mortality and comorbidities. Life-threatening conditions included heart diseases of varying complexity, but reversible heart diseases amenable to curative treatment were given less weight than irreversible heart diseases or those requiring palliative care. Prognosis and treatment decisions were made by a cardiologist. Genetic or environmental anomalies or anomalies of unknown etiology that result in functional impairment during fetal development were considered important indications for palliative care. The same applied to severe central nervous system conditions that led to disrupted brain activity and difficult-to-control seizures. Hypoxic-ischemic encephalopathy, genetic syndromes, epidermolysis bullosa, cystic fibrosis, severe osteogenesis imperfecta, and metabolic conditions with limited life expectancy, in addition to severe conditions such as trisomy 13 and 18, severe pulmonary hypoplasia with an oxygenation index ≥ 25.7, and skeletal dysplasia, were considered strong indicators for palliative care by the specialized team. The use of vasoactive drugs such as dobutamine, norepinephrine, dopamine, vasopressin, epinephrine, and milrinone was also considered. The need for ventilatory support, including invasive mechanical ventilation, was assessed by the ratio between arterial oxygen pressure and inspired fraction of oxygen (PaO_2_/FiO_2_). Lower values of this parameter indicate compromised ventilation and greater dependence on support.

**Table 1:** Palineo score

		Points attributed
Viability - gestational age score		
	< 25 weeks	10
	25 1/7 - 28 weeks	7
	28 1/7 - 30 weeks	4
	Above 30 weeks	0
Birth weight		
	< 500	10
	500 - 749g	7
	750 - 1,000g	3
	Above 1,000g	0
Limiting or life-threatening condition score		
	Reversible heart disease	2
	Irreversible heart disease	10
	Organic structural anomalies	8
	Severe central nervous system anomalies	10
	Skeletal dysplasia	10
	Hypoxic ischemic encephalopathy	8
	Diaphragmatic hernia	9
	Severe pulmonary hypoplasia	10
	Trisomy 13	10
	Trisomy 18	10
	Bullous epidermolysis	6
	Cystic fibrosis	5
	Severe osteogenesis imperfecta	6
	Other genetic syndromes	7
	Metabolic conditions with low life expectancy	6
Clinical conditions score		
	5-minute Apgar < 5	10
	Renal failure with dialysis indication	9
	Liver failure	5
	Grade III periventricular hemorrhage	7
	Grade IV periventricular hemorrhage	10
	Leukomalacia	6
	Pulmonary hemorrhage	6
	Previous cardiopulmonary arrest for more than 20 minutes	8
Use of vasoactive drugs		
	Dobutamine	4
	Norepinephrine/vasopressor	6
	Epinephrine	8
	Dopamine	2
	Milrinone	1
Ventilatory support		
	Need for invasive MV (PaO_2_/FiO_2_ ≥ 300)	3
	Need for invasive MV (PaO_2_/FiO_2_ = 200 - 300)	5
	Need for invasive MV (PaO_2_/FiO_2_ < 200)	10
	Need for noninvasive MV	3
	Need for oxygen therapy	1
	Dependence on oxygen therapy for more than 7 days	2
Devices score		
	Gastrostomy	5
	Tracheostomy	5

MV - mechanical ventilation.

A reduced PaO_2_/FiO_2_ ratio suggests an increase in the alveolar-arterial oxygen gradient, reflecting the presence of disorders such as refractory hypoxemia, alveolar-capillary membrane dysfunction and increased intrapulmonary shunt. The need for noninvasive mechanical ventilation regardless of the duration of use or oxygen therapy, especially when used for more than 7 days, is associated with several complications, mainly oxidative stress and lung inflammation. The main harms include bronchopulmonary dysplasia, retinopathy of prematurity, brain injury, systemic toxicity and impact on lung development.

Artificial means of ventilation and feeding (tracheostomy and/or gastrostomy) also indicate the need for palliative care due to reduced functionality, such as impaired swallowing, airway protection and breathing.

Unfavorable clinical conditions such as severe neonatal asphyxia (five-minute Apgar score less than or equal to 5), renal failure requiring dialysis, liver failure, grade III or IV intraventricular hemorrhage, periventricular leukomalacia, pulmonary hemorrhage, and previous cardiorespiratory arrest lasting more than 20 minutes were considered indicators of palliative care.

Finally, the experts classified the newborns into three groups based on the sum of the points attributed to the variables on the Palineo scale (Table 1): (I) Palliative intent, when newborns were admitted to the neonatal intensive care unit owing to a life-threatening condition that could be reversed and managed with curative treatment and without the need for prolonged care. In this group, physical, spiritual, and psychosocial care for the child and family can be provided by any health care professional; (II) Associated palliative care, when newborns have a life-limiting illness with a major impact on neuromotor development and possible dependence on assistive technology, artificial feeding, and airway management that cannot be cured but has the possibility of survival. This type of care should be provided by a multidisciplinary pediatric palliative care team; (III) Specialized palliative care, when newborns have genetic syndromes and/or irreversible clinical conditions, limited fetal viability, and dependence on advanced life support, with a high chance of developing serious sequelae and disabilities, as well as serious illnesses.

The newborns were then evaluated according to these criteria by other health professionals working in the neonatal intensive care unit, including doctors, speech therapists, physiotherapists, psychologists, nutritionists and occupational therapists, all trained in the Palineo score.

The newborns were then reevaluated by the same professionals until the outcome was achieved through a systematic checklist to assess the health status of the newborns. The total points for each newborn were then recorded using Palineo scoring.

The number of assessments of each patient varied according to the length of hospital stay. The first score was based on data collected on the third day of life to allow newborns to stabilize and adapt to the external environment. Information on the second score was collected on the seventh day of hospitalization, with subsequent scores collected weekly until discharge, death, or transfer of the newborn.

After application of the score, the data were compared with the expert assessments to evaluate the accuracy of the tool. The patient's care classification could be modified according to changes in their clinical status.

The Palineo score is available free of charge at https://www.score-palineo.com.

Fleiss’ kappa coefficient (κ) was used to evaluate the level of agreement among the experts regarding the classification of patients on the basis of their palliative care needs. Values greater than 0.75 were considered to represent acceptable agreement beyond chance.^(9)^ Subsequently, the assumptions of normal data distribution and homogeneity of variances of the variables were checked using the Kolmogorov-Smirnov test and Levene's test, respectively. Gestational age, scores for neonatal acute physiology (SNAP), birth weight, 1-minute Apgar score, 5-minute Apgar score, and the Palineo score were compared across the three palliative care groups (palliative intent, associated palliative care, and specialized palliative care) using the Kruskal-Wallis test, followed by the Mann-Whitney U test to identify significant differences where applicable. The chi-square test was used to compare the frequency of patients assigned to each palliative care approach between the initial and final application of the score. Subsequently, receiver operating characteristic (ROC) curves were plotted to identify the discriminant value among the palliative care approaches. Two independent analytical methods were employed to assess the three palliative care approaches dichotomously. The first analysis established discriminant values for patients categorized under palliative intent or associated palliative care, both at the initial application of the score and at the final scoring. Similarly, the second analysis estimated discriminant values for patients classified under associated palliative care or specialized palliative care. The accuracy of the discriminant values was then verified by sensitivity tests (true positive rate) and specificity (true negative rate), the construction of ROC curves, and analysis of the area under the curve (AUC) and the corresponding 95% confidence intervals (95%CI). The accuracy of the discriminant value was determined by the AUC and classified as "perfect" (AUC = 1), "exceptional" (0.9 ≤ AUC < 1), "excellent" (0.8 ≤ AUC < 0.9), "acceptable" (0.7 ≤ AUC < 0.8), or "poor" (AUC < 0.7). AUC values ≤ 0.5 were deemed statistically comparable to those of random classification. All analyses were performed using the Predictive Analytics Software (PASW) statistics 18.0 software (SPSS Inc., Chicago, USA), with the significance level set at 5% (P < 0.05).

## RESULTS

A total of 191 newborns were admitted to the neonatal ICU during the study period and considered eligible for the study. All of them had at least one life-threatening condition requiring treatment in a critical care setting. Three extremely premature newborns were excluded from the sample because they died within the first 72 hours after admission.

The kappa coefficient revealed a high level of agreement among the experts regarding the classification of patients on the basis of their palliative care needs (κ = 0.85).

Among the 188 active participants, patients classified under associated palliative care and specialized palliative care, had lower gestational age (p = 0.003 and p = 0.061), birth weight (p = 0.001 and p = 0.040), 1-minute Apgar (p < 0.001 and p = 0.13), and 5-minute Apgar (p < 0.001 and p = 0.003) and higher SNAP (p < 0.001 and p = 0.018) and Palineo scores (p < 0.001 and p = 0.001) than those classified under palliative intent. Furthermore, patients classified under associated palliative care had a lower Palineo score (p = 0.005) than those classified under specialized palliative care (Table 2).

**Table 2:** Characterization of patients after birth

Variables	Palliative intent (n = 150)	Associated palliative care (n = 34)	Specialized palliative care (n = 4)	p value
Gestational age (weeks)	34 (31 - 36)	28 (26 - 36)*	26 (36 - 4)	0.003
SNAP (points)	9 (0 - 21)^a^	28 (12 - 58)^b^ *	61 (58 - 2)^c^ *	< 0.001
Birth weight (g)	1,768 (1,289 - 2,666)	970 (670 - 2,190)*	683 (2,190 - 4)*	0.001
1-minute Apgar (points)	7 (6 - 8)	3 (2 - 5)*	5 (5 - 4)*	< 0.001
5-minute Apgar (points)	8 (8 - 9)	7 (5 - 8)*	7 (8 - 4)*	< 0.001
Palineo score	3 (1 - 10)	27 (23 - 31)*	40 (31 - 4)* †	< 0.001

a n = 133;

b n = 23;

c n = 2;

* significant difference from palliative intent approach (p < 0.01);

† significant difference from associated palliative approach (p < 0.01). Data are presented as median (interquartile range).

### Changes in patient classification

A significant change (p < 0.001) was observed in the classification of patients in terms of palliative care between the initial and final application of the Palineo score. At the end of the study, 2.0% and 1.3% of patients categorized under palliative intent in the initial application were reclassified to associated palliative care and specialized palliative care, respectively. Those patients initially classified under associated palliative care also experienced changes in classification: 41.2% were reclassified to palliative intent, and 26.5% were reclassified to specialized palliative care. On the other hand, all of the patients classified under specialized palliative care retained their initial classification (Table 3).

**Table 3:** Changes in patients’ classification between the first score application and the final application

First application	Final application		
	Palliative intent (n = 159)	Associated palliative care (n = 14)	Specialized palliative care (n = 15)
Palliative intent (n = 150)	145 (96.7)	3 (2.0)	2 (1.3)
Associated palliative care (n = 34)	14 (41.2)	11 (32.3)	9 (26.5)
Specialized palliative care (n = 4)	0 (0.0)	0 (0.0)	4 (100.0)

Data are presented as absolute and relative frequencies (%).

### Identification of discriminant values of the Palineo score

**Figure 1:**
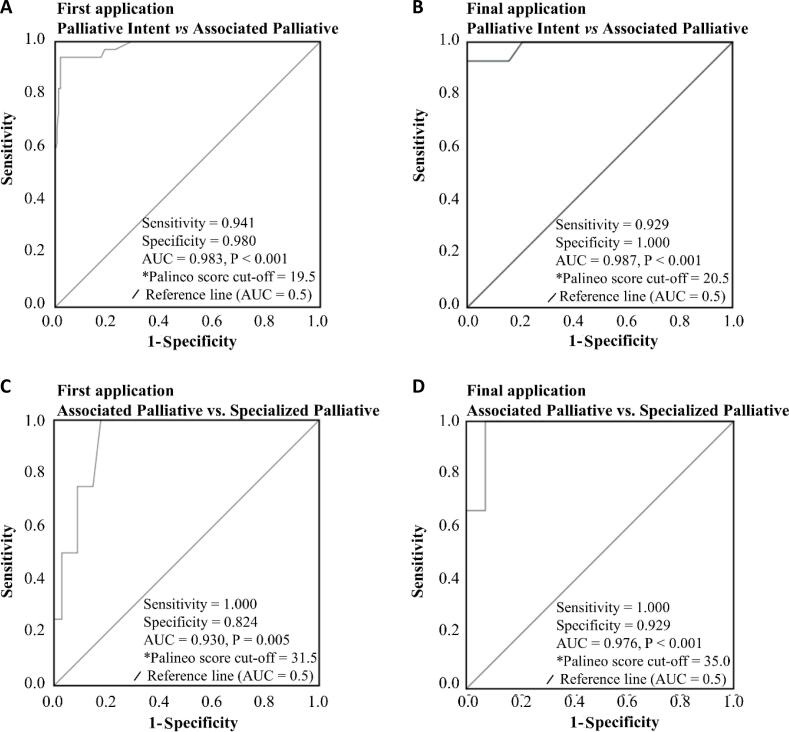
Receiver operating characteristic curve and discriminant cutoff value for palliative care classification on the basis of the Palineo score in the first and final applications.

The discriminant values of the Palineo score between patients classified under palliative intent or associated palliative care showed exceptional accuracy at the initial application of the score (AUC = 0.983; 95%CI = 0.963 - 1.00; p < 0.001; discriminant value = 19.5 points) and at the final application (AUC = 0.987; 95%CI = 0.962 - 1.00; p < 0.001; discriminant value = 21 points). The sensitivity values ranged from 92.9 - 94.1%, and the specificity values ranged from 98.0 - 100.0% (Figure 1A and 1B). The accuracy of the discriminant values between patients classified under associated palliative care and specialized palliative care was exceptional at the initial application of the score (AUC = 0.930; 95%CI = 0.841 - 1.00; p = 0.005; discriminant value = 31.5 points) and at the final application (AUC = 0.976; 95%CI = 0.925 - 1.00; p < 0.001; discriminant value = 35 points). The sensitivity was 100% in both cases, and the specificity ranged from 82.4 to 92.9% (Figure 1C and 1D).

AUC - area under the curve.

**Figure 2:**
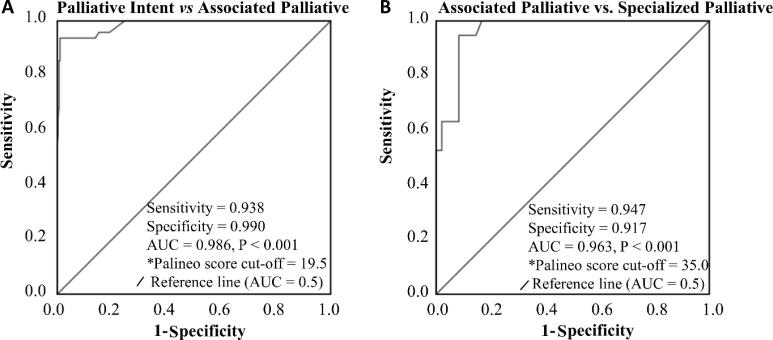
Receiver operating characteristic curve and discriminant cutoff value for palliative care classification based on the Palineo score for all applications (combined).

While several patients experienced reclassification into palliative care categories (Table 2) between the initial and final applications, the discriminant values of the Palineo score remained exceptional when all applications were analyzed together, with an AUC of 0.986 (95%CI = 0.971 - 1.000; p = 0.007; discriminant value = 19.5 points) for those classified under palliative intent and associated palliative care and an AUC of 0.963 between associated palliative care and specialized palliative care (AUC = 0.963; 95%CI = 0.925 - 1.000; p < 0.001; discriminant value = 35 points) (Figure 2).

AUC - area under the curve.

## DISCUSSION

Technological advances have increased the survival of newborns and the chances of detecting problems and risk factors in neonates.^(10)^ This group of newborns often requires admission to the neonatal ICU for treatment, given that they may present an unsatisfactory response and that their condition may progressively deteriorate, eventually threatening their lives.^(11)^

Unfortunately, a lack of knowledge or misunderstanding of palliative care still frequently has a negative impact on decision-making in health care. This misperception about the role of palliative care can lead to actions that exceed bioethical boundaries, resulting in inappropriate and ethically questionable practices, whether in maintaining, withdrawing, or not initiating life-sustaining measures.^(12)^

To date, no scale or scoring system for classifying neonatal palliative care needs has been described in the literature.

Given that eligibility for palliative care is inherently based on needs, it is essential to have an objective tool to clearly identify newborns who require such care. The Palineo score, a tool based exclusively on clinical criteria, can help identify and track these newborns. This score enables timely activation of specialized palliative care teams whenever necessary, ensuring that the family's concerns about the clinical status of the newborn are addressed and providing family members with emotional, psychological, and spiritual support.^(13)^ Eligibility and the score classification criteria were based on evidence from the literature.^(14–16)^

Care classifications could be determined not only by expertise but also by the Palineo score, which proved to be accurate enough to classify patient care according to the required complexity.

Given the challenges in recommending palliative care, the Palineo score was established with objective criteria that assess both the functional and clinical status of the newborn, thereby reducing potential bias associated with subjective professional opinions. Considering that 100% of neonatal ICU patients have at least one life-threatening condition, the primary indication was considered for the application of the score.

Our findings show that the Palineo score seems to be an effective tool for supporting decision-making on the basis of the patient's clinical status, whether it involves palliative intent, associated palliative care, or specialized palliative care. These findings consistently support the use of the Palineo score to identify newborns who require follow-up assessment by the palliative care team, comparing the scores with the actual events observed in clinical practice.

The cutoff values for the score, corresponding to the classification of palliative care, were as follows: up to 21 points for palliative intent, 22 to 31 points for associated palliative care, and above 32 points for specialized palliative care. These values were compared with those established by specialists: up to 19 points for palliative intent, 20 to 34 points for associated palliative care, and above 35 points for specialized palliative care. There was a slight variation in the score, but it managed to classify care from the beginning to the end of the study period.

Not all newborns need to be followed up by a specialized palliative care team. Many patients have less severe pathologies and conditions that are amenable to curative treatment. In these cases, which are classified as palliative intent, treatment can be provided by the care team.

When the clinical status of the newborn or infant deteriorates, the classification shifts to associated palliative care. In extremely severe cases, in which imminent death is expected, patients are classified under specialized palliative care, and they require follow-up by specialists.^(9)^

Note that all patients classified under specialized palliative care eventually died. Those patients classified under associated palliative care were discharged from the hospital, but all of them had some delay in neuropsychomotor development and neurological and/or pulmonary sequelae.

Some patients were transferred from associated palliative care to specialized palliative care, but none of them were transferred back to associated palliative care. However, patients initially classified under associated palliative care who showed clinical improvement were downgraded to palliative intent.

The utilization of a score for the classification of palliative care needs allows for the development of an advanced care plan with appropriate therapeutic measures. In the case of specialized palliative care, this approach allows family members to fulfill their wishes, such as religious rituals, birthday celebrations, and permission for additional visitors (e.g., grandparents and siblings), even in a controlled intensive care environment. It also allows parents to have the opportunity to hold their children regardless of their clinical stability and severity.

This score has several limitations. Despite the exceptional accuracy of the Palineo score in classifying palliative care into three categories, the discrimination values should be viewed with caution. The small sample sizes of the associated palliative care (n = 34) and specialized palliative care (n = 4) groups did not allow the assessment of wide variations in the scores of the tool. It is recommended that this study be replicated in larger samples to further validate the use of this tool, particularly in relation to large-scale discriminant values.

## CONCLUSION

The indication for palliative care for neonatal intensive care unit patients is routinely discussed in clinical practice. The Palineo score helps the neonatology team consider when palliative care is recommended and seek evaluation from the palliative care team as needed. This process optimizes patient follow-up, ensuring better physical, psychological, social, and spiritual support for newborns and their families, ultimately improving their quality of life. In light of our discriminant analysis, we suggest that the following points be used for the classification of palliative care using the Palineo score: < 20 points for palliative intent; 20 to 34 points for associated palliative care; and ≥ 35 points for specialized palliative care.

## References

[1] Galloway I, Roehr CC, Tan K (2024). Withdrawal and withholding of life-sustaining treatment (WWLST): an under recognised factor in the morbidity and mortality of periviable infants? – a narrative review. Transl Pediatr.

[2] Dombrecht L, Chambaere K, Beernaert K, Roets E, De Vilder De Keyser M, De Smet G (2023). Components of perinatal palliative care: an integrative review. Children (Basel).

[3] Spoljar D, Jankovic S, Vrkić D, McNamara G, Cuskovic M, Novak M (2025). Ethics and of life in pediatric and neonatal ICU's: a systematic review of recommendations. BM Palliat Care.

[4] Carter BS (2018). Pediatric palliative care in infants and neonates. Children (Basel).

[5] Leuthner SR, Cortezzo DE (2023). Editorial: advances in neonatal-perinatal palliative care. Front Pediatr.

[6] Barbosa SM, Molinari PC (2024). Auxílio na atuação do pediatra.

[7] Kain VJ, Chin SD (2020). Conceptually redefining neonatal palliative care. Adv Neonatal Care.

[8] Peralta D, Bogetz J, Lemmon ME (2023). Neurological conditions: prognostic communication, shared decision making, and symptom management. Semin. Fetal Neonatal Med.

[9] Bertaud S, Montgomery AM, Craig F (2023). Paediatric palliative care in the NICU: a new era of integration. Semin Fetal Neonatal Med.

[10] Gazzolla LP, Leite HV, Gonçalves GM (2020). Comunicando más notícias sobre malformações congênitas: reflexões bioéticas e jurídicas. Rev Bioét.

[11] Rent S, Bidegain M, Lemmon ME (2023). Neonatal neuropalliative care. Handb Clin Neurol.

[12] Vidal EI, Ribeiro SC, Kovacs MJ, Máximo da Silva L, Sacardo DP, Iglesias SB (2024). Position statement of the Brazilian Palliative Care Academy on withdrawing and withholding life-sustaining interventions in the context of palliative care. Crit Care Sci.

[13] Chong PH, Soo J, Yeo ZZ, Ang RQ, Ting C (2020). Who needs and continues to need paediatric palliative care? An evaluation of utility and feasibility of the Paediatric Palliative Screening scale (PaPaS). BMC Palliat Care.

[14] Rusalen F, Cavicchiolo ME, Lago P, Salvadori S, Benini F (2021). Perinatal palliative care: a dedicated care pathway. BMJ Support Palliat Care.

[15] Rent S, Lemmon ME, Ellestad S, Bidegain M (2023). The role of perinatal palliative care in fetal neurology. Am J Perinatol.

[16] Akyempon A, Aladangady N (2021). Neonatal and perinatal palliative care pathway: a tertiary neonatal unit approach. BMJ Paedriatr Open.

